# Preparation and Characterization of Acrylonitrile Butadiene Rubber Reinforced with Bio-Hydroxyapatite from Fish Scale

**DOI:** 10.3390/polym15030729

**Published:** 2023-01-31

**Authors:** Namthip Bureewong, Preeyaporn Injorhor, Saifa Krasaekun, Pawida Munchan, Oatsaraphan Waengdongbang, Jatuporn Wittayakun, Chaiwat Ruksakulpiwat, Yupaporn Ruksakulpiwat

**Affiliations:** 1 School of Polymer Engineering, Institute of Engineering, Suranaree University of Technology, Nakhon Ratchasima 30000, Thailand; 2 Research Center for Biocomposite Materials for Medical Industry and Agricultural and Food Industry, Suranaree University of Technology, Nakhon Ratchasima 30000, Thailand; 3 School of Chemistry, Institute of Science, Suranaree University of Technology, Nakhon Ratchasima 30000, Thailand

**Keywords:** acrylonitrile butadiene rubber, fish scale, bis(triethoxysilylpropyl)tetrasulfide, composite, compatibilizer

## Abstract

This work aims to enhance the mechanical properties, oil resistance, and thermal properties of acrylonitrile butadiene rubber (NBR) by using the *Nile tilapia* fish scales as a filler and using bis(triethoxysilylpropyl)tetrasulfide (TESPT) as a coupling agent (CA). The prepared fish scale particles (FSp) are B-type hydroxyapatite and the particle shape is rod-like. The filled NBR with FSp at 10 phr increased tensile strength up to 180% (4.56 ± 0.48 MPa), reduced oil absorption up to 155%, and increased the decomposition temperature up to 4 °C, relative to the unfilled NBR. The addition of CA into filled NBR with FSp at 10 phr increased tensile strength up to 123% (5.62 ± 0.42 MPa) and percentage of elongation at break up to 122% relative to the filled NBR with FSp at 10 phr. This work demonstrated that the prepared FSp from the *Nile tilapia* fish scales can be used as a reinforcement filler to enhance the NBR properties for use in many high-performance applications.

## 1. Introduction

*Nile tilapia* is a freshwater fish that finds public favor in consumption, which makes it popular in aquaculture. About 140,000 tons of *Nile tilapia* fish were produced in Thailand, and 23% of those were processed into fillets. That left about 2% of the *Nile tilapia* fish scales as waste [1,2,3]. Generally, fish scales are considered discarded waste from the food processing industry and are often disposed of in landfills, which pollute both soil and water resources [4,5]. Therefore, the development of fish scales into functional materials is interesting because it could reduce the impact of environmental pollution and increase their value. Normally, the fish scales are rich in hydroxyapatite, collagen, polysaccharide, and chitin, which contain the mineral elements magnesium, calcium, fluoride, and phosphorus [6,7]. Recently, fish scales have been used to produce various products, such as fertilizer and fillers for plastic and rubber industrials. Additionally, it also has a wide use in tissue engineering for biomedical applications [3,4]. Hydroxyapatite from fish scales has gained the attention of many researchers. Majhool et al. [8] have prepared hydroxyapatite from tilapia fish scales. They expected that it can be used as a potential filler in polymers. Similar to Prasad et al. [9], they have used hydroxyapatite from fish scales as a filler in polylactic acid (PLA) composites for use as fixation devices. Their work revealed that hydroxyapatite can be improved the wettability and thermal stability of PLA/hydroxyapatite composites.

Acrylonitrile butadiene rubber (NBR) is a synthetic rubber that has excellent resistance to solvents and oils due to the presence of polar cyanide groups in the NBR backbone. In addition, NBR is convenient to use in various industrial applications because of its moderate cost, processability, and heat resistance. The majority of NBR applications are used in petroleum and automobile industrials, such as fuel hoses, gaskets, oil seals, radiator hoses, v-belts, etc. [10,11]. However, NBR has drawbacks in tensile strength, flexibility and flame resistance, etc., that restrict its potential to be used in many applications requiring high performance [10,11,12,13,14,15]. Several researchers have recently been fabricating elastic composites, especially NBR-based nanocomposites, not only to improve the mechanical properties of NBR but also to enhance the resistance to solvents, oils, and heat [13,16,17].

However, although the addition of nanofillers to NBR has several advantages, as mentioned, it also has some disadvantages, such as the ability to agglomerate due to the filler–filler interactions, which limits the potential of the filler to improve the performance of NBR vulcanizate. Therefore, the addition of coupling agents could be an alternative way to reduce these problems because it would reduce the filler–filler interactions and enhance the filler–rubber interactions, which would improve the overall polymer composite properties [18,19]. Normally, bis(triethoxysilylpropyl)tetrasulfide (TESPT) is typically the coupling agent (CA) that is widely used in the rubber industry due to its low cost, availability, and the simplicity of the process [20]. TESPT is a bifunctional compound which is composed of two functionally active end groups. It can act as a bridge between filler and rubber via chemical linkages in the sulfur vulcanization, so enhancing of the rubber–filler interaction occurred [21].

In this work, alkali heat treatment was used to prepare *Nile tilapia* fish scales as fish scale particles (FSp). The method was based on previous research [22]. The particle size of FSp was expected to decrease with increasing treatment time and can be used as a filler in NBR. The characteristics of FSp were characterized using an X-ray diffractometer (XRD), an energy dispersive X-ray spectrometer (EDS), Fourier transform infrared spectroscopy (FTIR), a nitrogen adsorption–desorption analyzer, and a field emission scanning electron microscope (FE-SEM). The effects on the cure characteristics, mechanical properties, morphological properties, oil resistance, and thermal properties of the filled NBR with FSp at different contents (0, 5, and 10 phr) were investigated. Furthermore, the effects of CA on NBR samples with optimal FSp content were compared. NBR composites filled with FSp were expected to provide the oil resistance properties. This is a novelty of the work.

## 2. Materials and Methods

### 2.1. Materials

*Nile tilapia* fresh fish scales of approximately 3 kg were collected from the local market in Nakhon Ratchasima, Thailand. Acrylonitrile butadiene rubber (NBR) with the trademark NANCAR^®^ 3345 was supplied by NANTEX Industry Co., Ltd. (Kaohsiung, Taiwan). Bis(triethoxysilylpropyl)tetrasulfide (TESPT) with the product ID KBE-846 was supplied by Shin-Etsu Chemical Co., Ltd. (Tokyo, Japan). Hydrochloric acid (HCl) with AR grade and 37% purity was supplied by RCI Labscan Co., Ltd. (Bangkok, Thailand). Sodium hydroxide (NaOH) with RPE grade and 99% purity was supplied by Carlo Erba (Milan, Italy). Stearic acid (SA), zinc oxide (ZnO), Di(benzothiazol-2-yl)disulfide (MBTS), N-Cyclohexylbenzothiazole-2-sulfenamide (CBS), and sulfur (S) were supported by Chemical Innovation Co., Ltd. (Bangkok, Thailand).

### 2.2. Preparation of Fish Scale Particles (FSp) from Fresh Fish Scales

The method for preparing FSp was adapted from Kongsri et al. and Injorhor et al. [22,23]. The collected fresh fish scales were washed with tap water several times to remove dirt, and dried using a hot-air oven at 60 °C for 12 h to obtain the dried fish scales. The protein and fat on the surface of the dried fish scales were removed using HCl solution at 0.1 M under stirring for 2 h at room temperature. Then, the removed fish scales were filtered, washed with DI water until they reached pH = 7, and dried at 60 °C using a hot-air oven. The prior removed fish scales were alkali heat treated with 50% (*w*/*v*) of NaOH solution at 70 °C for 7 h. Afterwards, the slurry of fish scales was filtered, washed with DI water until it reached pH = 7, and dried using a hot-air oven at 60 °C. The FSp were then obtained.

### 2.3. Characterizations of FSp

The crystalline phase compositions and diffraction lines of FSp were analyzed by an X-ray diffractometer (XRD, D2 PHASER, Bruker, Billerica, MA, USA) with Cu Kα radiation source operated at 30 kV and current 10 mA. The 2θ range carried out was between 10 and 60 degrees.

The elemental compositions of FSp were analyzed by an energy dispersive X-ray spectrometer (EDS, EDAX Genesis 2000, AMETEK, Inc., Berwyn, PA, USA) in a scanning electron microscope (SEM-EDS, JSM-6010LV, JEOL, Tokyo, Japan).

The functional groups of FSp were analyzed by Fourier transform infrared spectroscopy (FTIR, TENSOR 27, Bruker, Billerica, MA, USA). The sample was mixed with potassium bromide (KBr) using agate mortar and pressed into a disk to obtain the test specimen with a smooth surface for transmittance measurement. The wavenumber range of 4000 to 400 cm^−1^ with resolution of 4 cm^−1^ and number of scans of 64 were used to collect data.

The characteristics of FSp in terms of BET surface area, total pore volume and particle size were determined from nitrogen adsorption–desorption analysis, which was performed on BELSORP-mini II (MicrotracBEL, Osaka, Japan). The sample was degassed at 160 °C for 6 h before analysis. The microstructure of FSp was acquired using a field emission scanning electron microscope (FE-SEM, AURIGA, Carl Zeiss, Oberkochen, Germany) at 3 kV. The samples were sputtered with gold for 3 min at 10 mA beforehand.

### 2.4. Preparation of NBR/FSp and NBR/FSp/CA Composites

The NBR/FSp and NBR/FSp/CA composites were prepared by compounding using an internal mixer at 70 °C with roller speed at 40 rpm and vulcanizing using a compression molding machine at 160 °C with an optimal cure time of each compound that was determined using a moving die rheometer (MDR). First, the NBR was masticated for 4 min, followed by the addition of different FSp contents (0, 5, and 10 phr), CA, and vulcanizing agent for 2 min. Then, the accelerators and activators were added separately for 1 min. The sample codes and compositions of NBR/FSp and NBR/FSp/CA composites are listed in Table 1.

### 2.5. Characterizations of NBR/FSp and NBR/FSp/CA Composites

The cure characteristics, such as minimum torque, maximum torque, scorch time, and optimal cure time of NBR/FSp and NBR/FSp/CA composites, were determined using an MDR (M-2000AN, GOTECH, Taichung, Taiwan) according to ASTM D2084 with a temperature at 160 °C. The cure rate index of NBR/FSp and NBR/FSp/CA composites were calculated by following the equation:

Cure rate index = 100/(optimal cure time − scorch time)(1)

The modulus at 100% elongation (M100), modulus at 300% elongation (M300), tensile strength, and percentage of elongation at break of NBR/FSp and NBR/FSp/CA composites were measured according to ASTM D412 using a universal testing machine (UTM, Model:5565, INSTRON, Norwood, MA, USA) with a load cell of 5 kN and crosshead speed of 500 mm/min.

The hardness of NBR/FSp and NBR/FSp/CA composites were measured according to ASTM D2240 using a hardness tester (HPE II, Bareiss, Stouffville, ON, Canada) with the Shore A test method.

The secondary electron images of NBR/FSp and NBR/FSp/CA composites were acquired using a field emission scanning electron microscope (FE-SEM, AURIGA, Carl Zeiss, Oberkochen, Germany). The tensile fracture surfaces of NBR/FSp and NBR/FSp/CA composites were coated with gold before the SEM observation.

The oil resistance in terms of change in mass percentage of NBR/FSp and NBR/FSp/CA composites were performed according to ASTM D471 by immersing the standard specimens in toluene for 22 h. The equation that was used to calculate the change in mass percentage of NBR/FSp and NBR/FSp/CA composites is:

ΔM (%) = [(M_2_ − M_1_)/M_1_] × 100 (2)

where ΔM is the change in mass (%), M_1_ is the initial mass of the specimen in air (g), and M_2_ is the mass of specimen in air after immersion (g).

The thermogravimetric analyzer (TGA, TGA/DSC 1, METTLER TOLEDO, Greifensee, Switzerland) was used to analyze the thermal decompositions of NBR/FSp and NBR/FSp/CA composites. The specimens were placed in an alumina pan and heated from room temperature up to 650 °C under nitrogen with a heating rate of 10 °C/min and a gas flow rate of 20 mL/min.

## 3. Results and Discussion

### 3.1. Characterizations of FSp

**Figure 1:**
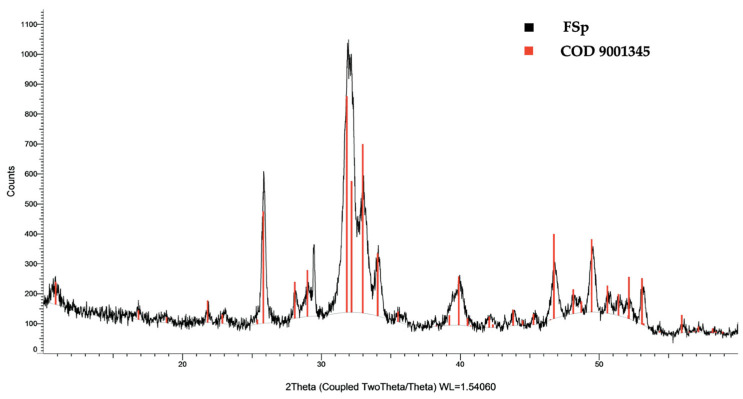
XRD pattern of FSp compared with COD 9001345.

The XRD pattern of FSp is represented in Figure 1. The sample consisted entirely of the hydroxyapatite phase with defined peaks following the Crystallography Open Database (COD 9001345) and similar to the standard of JCDPS 00-009-0432 [24] without other phases. The characteristic peaks of the FSp are similar to the synthetic hydroxyapatite of Pon-On et al. [25] and the fish scale nano-hydroxyapatite of Kongsri et al. [23]. It was confirmed that the obtained FSp are a type of hydroxyapatite, which is a bio-material.

**Figure 2:**
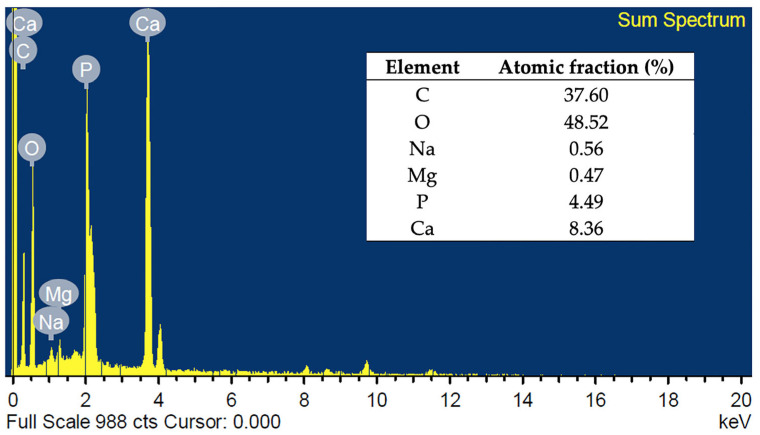
Elemental analysis by EDS of FSp.

The elemental compositions and EDS spectra of FSp are shown in Figure 2. It confirms that the major constituents are calcium (Ca), phosphorous (P), and oxygen (O). Moreover, the typical presence of sodium (Na) and magnesium (Mg) are significant factors in bone and tooth growth [26]. However, the Ca/P of FSp is 1.86, which is higher than the stoichiometric ratio of 1.67 for stoichiometric hydroxyapatite because of the presence of carbonate (CO_3_^2−^) ions that substitute phosphate (PO_4_^3−^) in the hydroxyapatite structure (B-type hydroxyapatite) [25].

**Figure 3:**
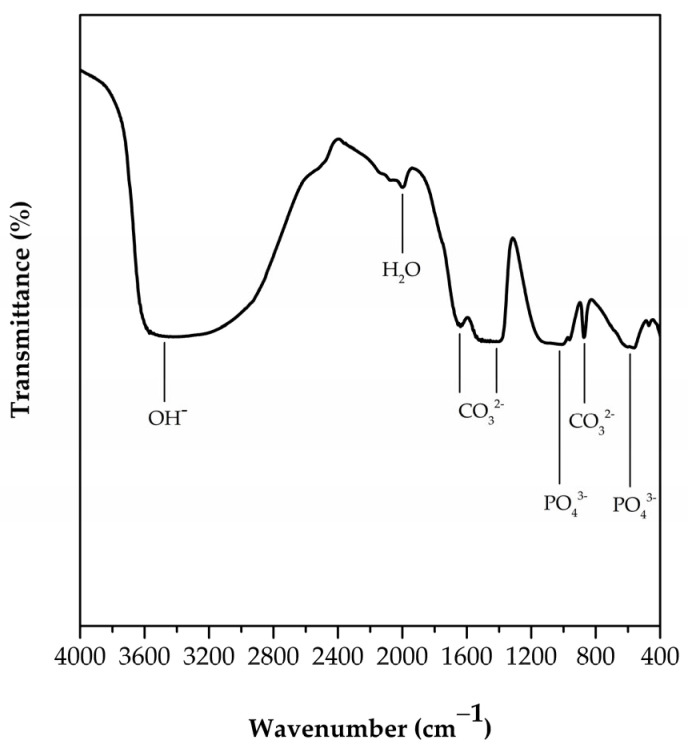
FTIR spectrum of FSp.

The functional groups of FSp are shown in Figure 3. The broad band around 3500 cm^−1^ corresponds to the OH^−^ stretching vibration of adsorbed water. The bands at 1471 and 1415 cm^−1^ correspond to the asymmetric stretching vibration of the CO_3_^2−^ band, and a band at 873 cm^−1^ corresponds to the bending vibration of CO_3_^2−^ [23,27]. The presence of CO_3_^2−^ bands indicated that some PO_4_^3−^ groups were replaced with CO_3_^2−^ groups. These results confirmed the FSp are B-type hydroxyapatite, which corresponds to the EDS result and with Kongsri et al. [23]. In addition, the strong band at 1043 cm^−1^ corresponds to the stretching vibration of PO_4_^3−^, and the sharp bands at 601 and 563 cm^−1^ correspond to the degenerate bending vibrations of PO_4_^3−^ in a hydroxyapatite structure.

**Figure 4:**
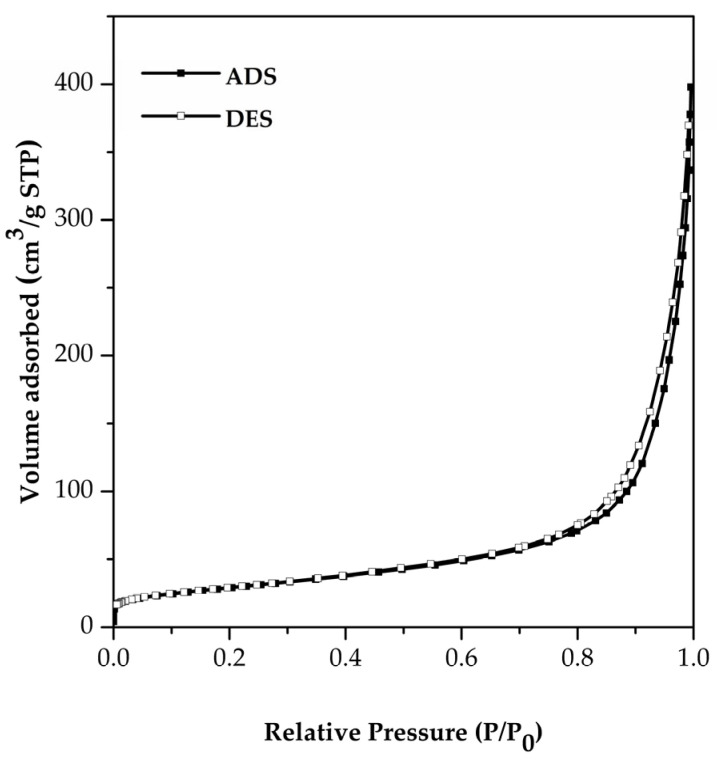
Physisorption isotherm of FSp.

The adsorption–desorption isotherm of FSp is represented in Figure 4 and the information on FSp analysis in terms of BET surface area and total pore volume are listed in Table 2. The isotherm shape shows unrestricted monolayer–multilayer adsorption up to high P/P_0_ without the final saturation plateau. Therefore, the isotherm of FSp is fitted to the second (II) type of the IUPAC classification given by nonporous adsorbents [28,29]. The BET surface area of FSp shows a higher value as compared to the extracted hydroxyapatite from carp fish that was reported by Muhammad et al. [30]. In addition, the BET surface area of FSp shows a higher value than the commercial hydroxyapatite in these reports [23,30]. For use as filler in composite materials, the high surface area of FSp has an advantage in terms of greater interactions with the matrix. It was expected to be a bio-filler for improving NBR composites.

**Figure 5:**
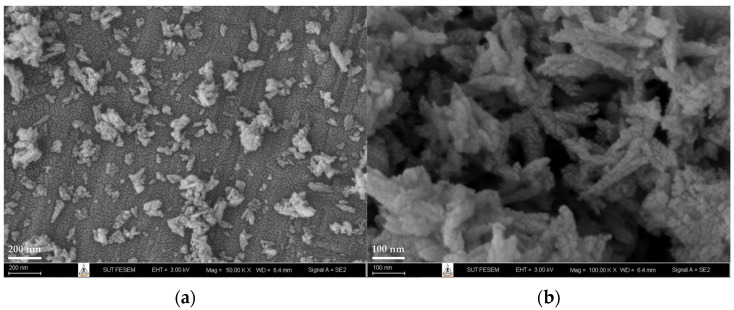
FE-SEM images of FSp at (**a**) ×50,000 and (**b**) ×100,000 magnification.

The microstructure of FSp is shown in Figure 5. The FE-SEM images show the FSp in a rod-like shape with some agglomerates of FSp due to the static force between the FSp. However, the particle size of the FSp is in the range of the nanoscale.

### 3.2. Characterizations of NBR/FSp and NBR/FSp/CA Composites

The cure characteristics such as minimum torque, maximum torque, scorch time, optimal cure time, and cure rate index of NBR/FSp and NBR/FSp/CA composites are listed in Table 3. Normally, the minimum torque is related to the viscosity of the rubber compound, while the maximum torque is related to the rigidity of the rubber vulcanizate. In the case of the addition of filler to rubber, these properties are also related to the nature of the filler. In addition, the scorch time is the time with no crosslinks in the rubber compound, which is an important parameter for the safe processing of rubber in molds. The optimal cure time is another important parameter that determines the time required to produce the rubber products [15,31,32,33,34,35]. The value of minimum torque of NBR-5FSp tends to increase as compared to unfilled NBR because the dispersion of FSp at 5 phr reduces the mobility of the macromolecular chains of NBR, which causes the viscosity of the compound to increase [15,32,34]. Meanwhile, the value of minimum torque of NBR-10FSp decreases as compared to unfilled NBR and NBR-5FSp because the addition of FSp at this amount has agglomerated in the NBR matrix. The values of maximum torque of the filled NBR with FSp are higher than unfilled NBR because the stiffness of the FSp increases the rigidity of composite vulcanizates. The values of scorch time and optimal cure time of filled NBR with FSp decrease as compared to unfilled NBR because the mineral content of FSp acts as an activator for composite vulcanizates [4,34]. Meanwhile, the NBR-10FSp slightly decreases the values of scorch time and optimal cure time as compared to NBR-5FSp because the system of filled NBR with increasing FSp content becomes more heated from the filler friction that affects the increased degree of curing. These results resemble the cure characteristics of filled rubbers with prepared hydroxyapatite that were reported by Nihmath and Ramesan [15,34]. Therefore, the use of FSp as a filler in NBR is an alternative idea for producing NBR composite because it can reduce the optimal cure time of NBR to obtain the product in a shorter time. However, the NBR-10FSp-CA shows the value of minimum torque decreasing as compared to filled NBR because the CA acts as a lubricant, which causes the viscosity of the compound to decrease. Moreover, the value of the optimal cure time of NBR-10FSp-CA tends to increase as compared to filled NBR because the CA coats on the surface of FSp, which reduces their activator activity, which causes the optimal cure time of the composite vulcanizate to increase.

**Figure 6:**
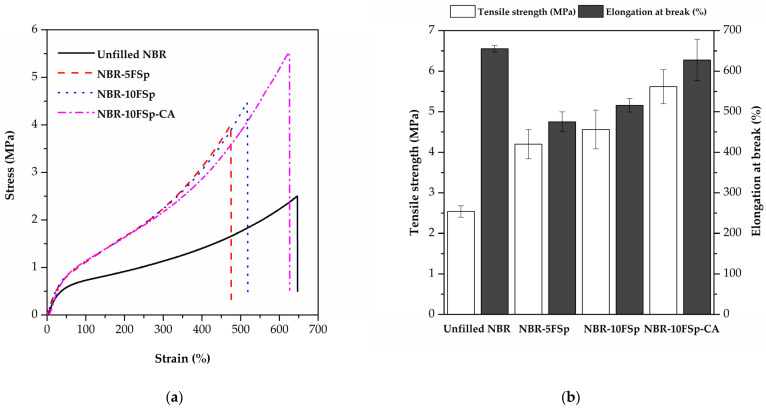
Mechanical properties of NBR/FSp and NBR/FSp/CA composites showing (**a**) Stress–strain curves and (**b**) Tensile strength and percentage of elongation at break.

**Figure 7:**
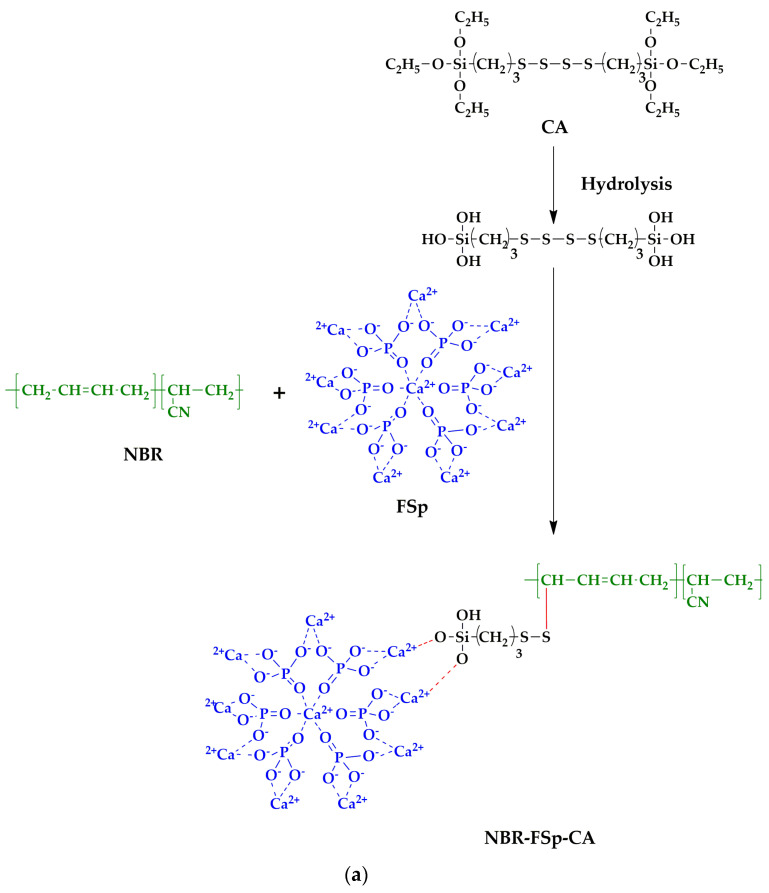

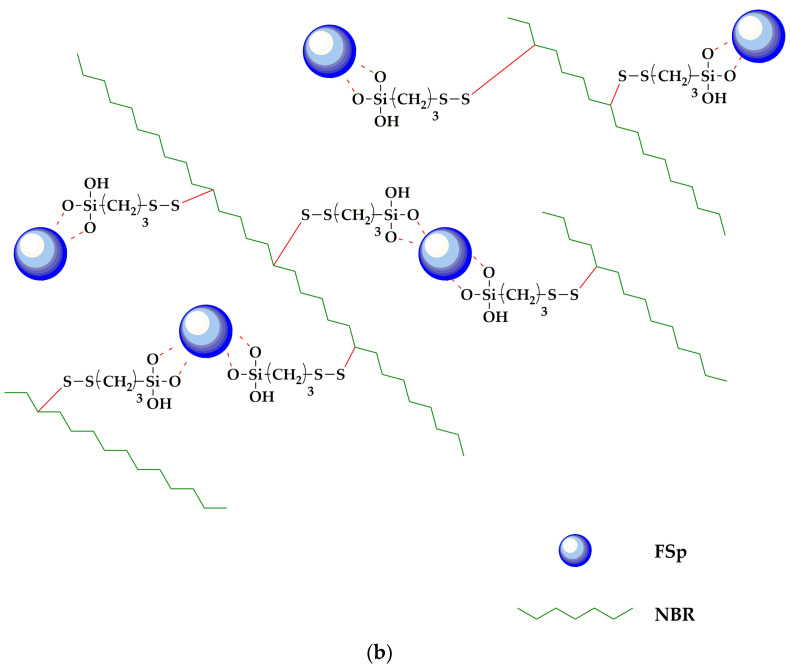
(**a**) Schematic representation of NBR/FSp/CA interactions and (**b**) Sketch of the interface interaction between FSp, CA, and NBR chains.

Figure 6 represents the mechanical properties of NBR/FSp and NBR/FSp/CA composites, and Table 4 shows the values of the mechanical properties of NBR/FSp and NBR/FSp/CA composites in terms of M100, M300, tensile strength, percentage of elongation at break, and hardness. It is well known that the characteristics and dispersion of filler are directly related to the properties of the polymer composite. Additionally, the area under the stress–strain curves is related to the toughness of the polymer. In general, the mechanical properties depend on the nature of the filler, dispersion, and the interaction between the filler and polymer matrix [4,15,34,36]. The modulus and tensile strength of filled NBR with FSp show increased values as compared to unfilled NBR because of the interactions between FSp and the NBR matrix, which improve the fracture resistance of NBR composites. However, the percentage of elongation at the break of filled NBR with FSp shows decreased values as compared to unfilled NBR because the addition of FSp restricts the molecular motions of the NBR matrix, which reduces the elasticity of NBR. When compared to the filled NBR with FSp at 5 and 10 phr, the NBR-10FSp shows higher values of tensile strength and percentage of elongation at break that resemble the mechanical properties reported by Akbay et al. [4]. According to this research, the tensile strength and percentage of elongation at break of the rubber were increased by increasing the fish scale content, because the calcium oxide (CaO) content of fish scale acts as a vulcanizate activator that improved the mechanical strength and flexibility of the rubber composite. Meanwhile, the hardness values of filled NBR increase with increasing FSp content because the stiffness of FSp improves the resistance to indentation of NBR composite, which resembles the hardness results in these reports [15,34]. Moreover, the NBR-10FSp-CA shows increased values of tensile strength and percentage of elongation at break as compared to filled NBR because the CA enhances the chemical interaction between FSp and the NBR matrix, which assists the stress-transfer of NBR composite but has no effect on the modulus value. Figure 7a represents the schematic of NBR/FSp/CA interactions. The first step is CA hydrolysis, which generates the silanol groups (Si-OH) on the side chains of CA. The second step is that the Si-OH of CA and the Ca of FSp generate an ionic reaction together. In addition, the sulfur (S) of the CA and the carbon (C) on the NBR chains undergo a crosslinking reaction. Figure 7b represents a sketch of the interface interactions between FSp, CA, and NBR chains. These interactions are similar to these reports [19,37,38]. All results show that the modulus, tensile strength, and hardness of NBR are improved by adding the FSp with increasing contents. In addition, the tensile strength, percentage of elongation at break, and hardness of NBR composites are also improved by using a CA to obtain superior values.

**Figure 8:**
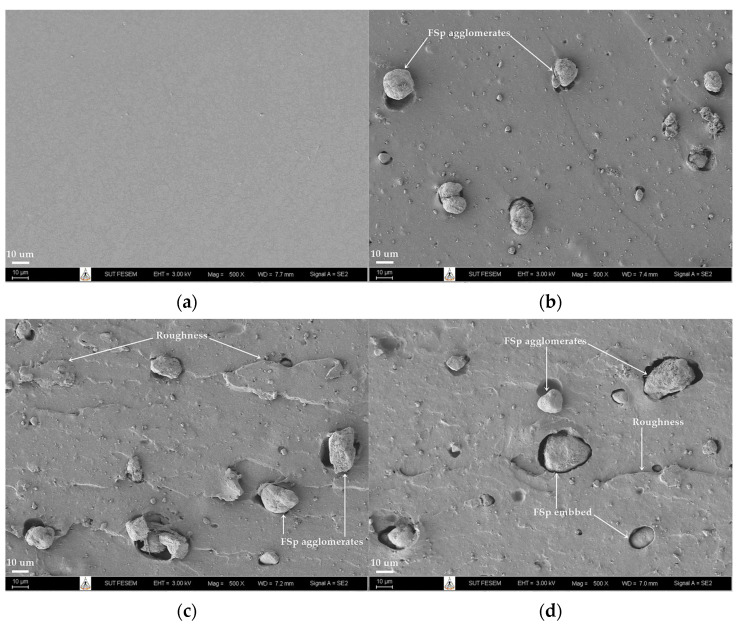
FE-SEM images of (**a**) Unfilled NBR, (**b**) NBR-5FSp, (**c**) NBR-10FSp, and (**d**) NBR-10FSp-CA composites.

Figure 8 presents the FE-SEM images of tensile fracture surfaces of the NBR/FSp and NBR/FSp/CA composites. Generally, the morphological properties of the polymer composite are necessary to report in order to understand the dispersion, compatibility, and characteristics of the filler after the mixing and forming processes [13,32,39]. The unfilled NBR shows the smooth fracture surface without the particles on the surface (Figure 8a). On the other hand, the filled NBR with FSp shows the dispersion of FSp with some agglomerates on the rough fracture surface (Figure 8b,c). These characteristics tend to increase with increasing FSp content in NBR (Figure 8c). In general, the dispersion of the filler should be uniform with no agglomerates in a polymer composite. Nevertheless, the mechanical properties in terms of modulus, tensile strength, and hardness of filled NBR increase with increasing FSp content. This indicated that the FSp are effective reinforcement fillers for improving the mechanical properties of NBR, although the FSp exhibit non-uniform dispersion and have some agglomeration in the NBR matrix. Additionally, the roughness on the fracture surface indicates the resistance to fracture of the polymer composite due to the good mechanical interlocking between filler and matrix [32]. That corresponds to the results of tensile strength and percentage of elongation at break of filled NBR, which increase with increasing FSp content. In the case of the addition of CA, the NBR-10FSp-CA shows the image of fracture surface with similar characteristics as compared to the NBR-10FSp. However, the FSp on the fracture surface of NBR-10FSp-CA tend to be more embedded in the NBR matrix than the FSp on the fracture surface of NBR-10FSp (Figure 8d), which affects the increased tensile strength and the percentage of elongation at break of the NBR-10FSp-CA. This indicated that the CA increases the reinforcement efficiency of the FSp and NBR matrix.

**Figure 9:**
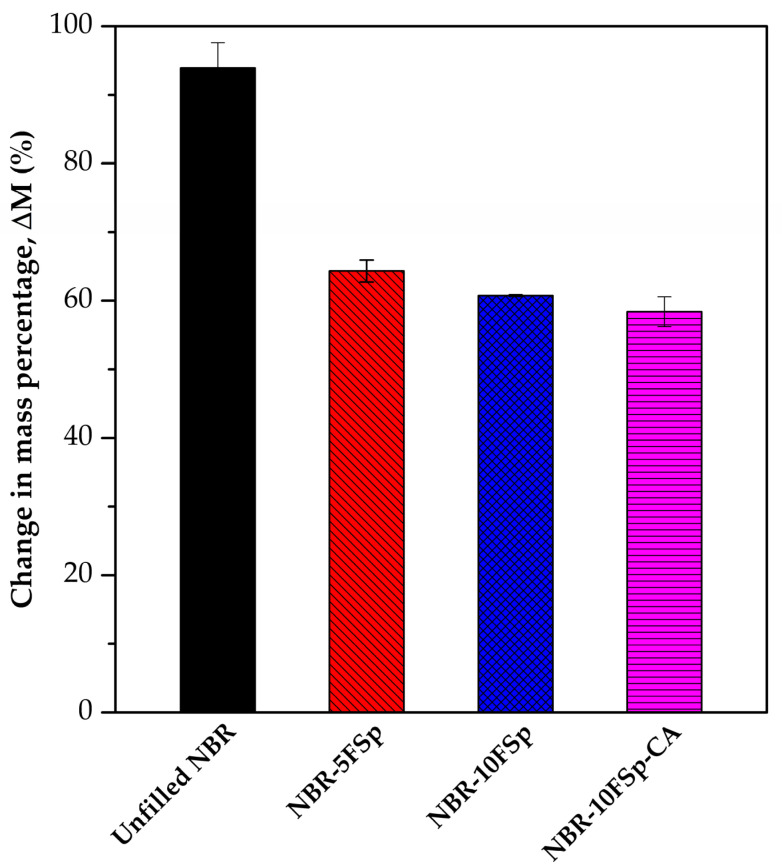
Oil resistance of NBR/FSp and NBR/FSp/CA composites.

Figure 9 depicts the oil resistance of NBR/FSp and NBR/FSp/CA composites in terms of change in mass percentage. The oil resistance is an important parameter for polymer products that are used in petroleum applications. In general, the oil resistance depends on the interactions between solvent and polymer, crosslink density, and crystallinity of polymer [15,34]. The oil resistance of NBR is enhanced by adding the FSp, because the intermolecular forces between FSp and NBR increase the energy required to separate the NBR molecules for the penetration of oil molecules, which affects the decreased swelling percentages of filled NBR with FSp. In addition, this result also depends on the content of FSp added, which shows a decrease in the swelling percentage with increasing FSp content in NBR. Meanwhile, the value of the swelling percentage of NBR-10FSp-CA is no different when compared to NBR-10FSp, which indicates that the CA has no effect on this property. The values of the swelling percentage of unfilled NBR, NBR-5FSp, NBR-10FSp, NBR-10FSp-CA are 93.91%, 64.33%, 60.73%, and 58.41%, respectively. Therefore, although it is well known that NBR is a synthetic rubber that has excellent resistance to solvents and oils, the filled NBR with increasing FSp content increases the oil resistance efficiency.

**Figure 10:**
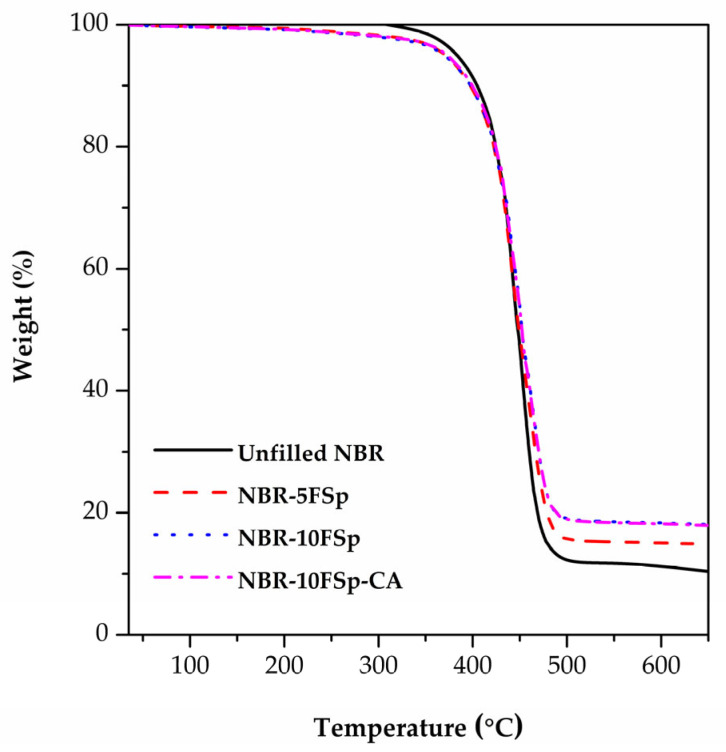
TGA thermograms of NBR/FSp and NBR/FSp/CA composites.

The TGA thermograms of NBR/FSp and NBR/FSp/CA composites are presented in Figure 10, and the thermal properties in terms of the initial degradation temperature (T_onset_), temperature at maximum weight loss level (T_max_), final degradation temperature (T_endset_), and residue percentages of NBR/FSp and NBR/FSp/CA composites are listed in Table 5, according to the TGA thermograms of NBR composites, which can divide the stages of weight loss into three stages: 35–350, 350–500, and 500–650 °C. The first stage shows a slightly decreased weight loss percentage in all samples, which is about 3% due to the volatile water. Meanwhile, the decomposition of organic compounds from filler and polymer is the reason for the maximum weight loss in all samples that is shown in the second stage. However, the values of T_onset_ of NBR tend to increase with increasing FSp content, and the results of T_max_ and T_endset_ also show this tendency. The reason for these results is that the decomposition temperature of FSp is higher than the NBR matrix, so the FSp act as a heat barrier during the thermal decomposition process of NBR composites, which resembles the thermal properties reported in these reports [13,40,41]. According to these studies, the thermal stability of the polymer was increased by increasing the prepared hydroxyapatite content. Additionally, due to the incomplete decomposition of organic compounds in the second stage, all samples exhibit a slightly decreased weight loss percentage in the third stage. At a temperature of 650 °C, the values of residue percentage of NBR increased with increasing FSp content due to the remaining inorganic compounds from FSp in NBR composites. Nevertheless, the NBR-10FSp and NBR-10FSp-CA show similar results in their thermal properties. Therefore, the thermal properties of NBR are increased by adding FSp with increasing content, but the CA has no effect on these results.

## 4. Conclusions

FSp were successfully obtained from *Nile tilapia* fish scales biowaste and used to prepared NBR/FSp composites. The obtained FSp are B-type hydroxyapatite with a rod-like shape. The FSp were the effective reinforcement filler in the NBR matrix because it enhanced the tensile strength, oil resistance, and thermal properties of NBR. Moreover, the scorch time and optimal cure time of NBR also reduced with increasing FSp content, resulting in a shorter time to obtain the NBR product. The addition of CA gave the best properties, because the CA enhanced the tensile strength and percentage of elongation at break of NBR-10FSp. The obtained NBR composites filled with FSp are expected to be used in sealing gadgets that can resist oil. In the future, the NBR composites will compare with other bio-fillers.

## Figures and Tables

**Table 1:** Sample codes and compositions of NBR/FSp and NBR/FSp/CA composites.

Materials (phr *)	Unfilled NBR	NBR-5FSp	NBR-10FSp	NBR-10FSp-CA
NBR	100	100	100	100
SA	2	2	2	2
ZnO	5	5	5	5
MBTS	1.5	1.5	1.5	1.5
CBS	0.5	0.5	0.5	0.5
S	1.5	1.5	1.5	1.5
FSp	-	5	10	10
TESPT	-	-	-	2

* phr = part per hundred of rubber.

**Table 2:** Information on FSp analysis.

Sample	BET Surface Area (m^2^/g)	Total Pore Volume (cm^3^/g)
FSp	104	0.50

**Table 3:** Cure characteristics of NBR/FSp and NBR/FSp/CA composites.

Samples	Minimum Torque (dNm)	Maximum Torque (dNm)	Scorch Time (min)	Cure Time (min)	Cure Rate Index (min^−1^)
Unfilled NBR	7.50	23.12	3:32	11:53	0.20
NBR-5FSp	7.86	30.25	1:32	2:38	1.52
NBR-10FSp	6.44	29.02	1:31	2:32	1.64
NBR-10FSp-CA	5.91	24.00	1:32	3:12	1.00

**Table 4:** Mechanical properties of NBR/FSp and NBR/FSp/CA composites.

Samples	M100 (MPa)	M300 (MPa)	Tensile Strength (MPa)	Elongation at Break (%)	Hardness (Shore A)
Unfilled NBR	0.73 ± 0.02	1.14 ± 0.06	2.54 ± 0.14	655.35 ± 8.07	35.40 ± 0.25
NBR-5FSp	1.15 ± 0.04	2.39 ± 0.13	4.20 ± 0.36	475.21 ± 24.37	37.90 ± 0.30
NBR-10FSp	1.14 ± 0.02	2.21 ± 0.06	4.56 ± 0.48	515.81 ± 16.68	38.33 ± 0.25
NBR-10FSp-CA	1.13 ± 0.03	2.21 ± 0.08	5.62 ± 0.42	627.56 ± 51.10	41.40 ± 1.14

**Table 5:** Thermal properties of NBR/FSp and NBR/FSp/CA composites.

Samples	T_onset_ (°C)	T_max_ (°C)	T_endset_ (°C)	Residue (%)
Unfilled NBR	410	445	475	10.91
NBR-5FSp	411	446	482	14.04
NBR-10FSp	412	449	483	17.47
NBR-10FSp-CA	413	449	483	17.23

## Data Availability

Not applicable.
